# Bruton's tyrosine kinase is essential for NLRP3 inflammasome activation and contributes to ischaemic brain injury

**DOI:** 10.1038/ncomms8360

**Published:** 2015-06-10

**Authors:** Minako Ito, Takashi Shichita, Masahiro Okada, Ritsuko Komine, Yoshiko Noguchi, Akihiko Yoshimura, Rimpei Morita

**Affiliations:** 1 Department of Microbiology and Immunology, Keio University School of Medicine, Shinjuku-ku, Tokyo 160-8582, Japan; 2 Japan Science and Technology Agency (JST), CREST, Chiyoda-ku, Tokyo 102-0075, Japan; 3 PRESTO (Precursory Research for Embryonic Science and Technology), Chiyoda-ku, Tokyo 102-0075, Japan

## Abstract

Inflammasome activation has been implicated in various inflammatory diseases including post-ischaemic inflammation after stroke. Inflammasomes mediate activation of caspase-1, which subsequently induces secretion of pro-inflammatory cytokines such as IL-1β and IL-18, as well as a form of cell death called pyroptosis. In this study, we report that Bruton's tyrosine kinase (BTK) is an essential component of the NLRP3 inflammasome, in which BTK physically interacts with ASC and NLRP3. Inhibition of BTK by pharmacological or genetic means severely impairs activation of the NLRP3 inflammasome. The FDA-approved BTK inhibitor ibrutinib (PCI-32765) efficiently suppresses infarct volume growth and neurological damage in a brain ischaemia/reperfusion model in mice. Ibrutinib inhibits maturation of IL-1β by suppressing caspase-1 activation in infiltrating macrophages and neutrophils in the infarcted area of ischaemic brain. Our study indicates that BTK is essential for NLRP3 inflammasome activation and could be a potent therapeutic target in ischaemic stroke.

## Abstract (web summary)

Activation of inflammasome contributes to several pathologies. Here, the authors show that Bruton's tyrosine kinase is essential for NLRP3 inflammasome activation, and that blocking it with the FDA-approved inhibitor ibrutinib limits tissue damage in a mouse model of ischaemic stroke.

## Graphical abstract

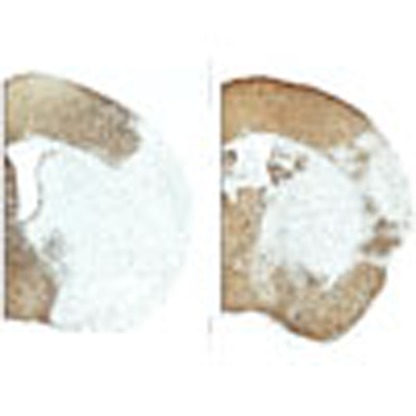

Stroke causes ischaemic brain injury, including brain infarction, which is a leading cause of neurological disability and death worldwide. At present, plasminogen activator (t-PA) is globally approved for the treatment of ischaemic brain injury, but the therapeutic time window of t-PA is only 4.5 h after stroke onset. There is a need for an efficacious therapy that can be administered beyond this time window^1^. Post-ischaemic inflammation is a hallmark of ischaemic stroke pathology^2^,^3^. Inflammatory cytokines from myeloid cells, such as interleukin (IL)-1β, tumour necrosis factor (TNF)-α and IL-23, and T-cell-derived cytokines, such as IL-17 and IL-21, promote brain tissue injury and are therefore potential targets for therapy after ischaemic stroke^4^,^5^,^6^. Pattern recognition receptors including toll-like receptors (TLRs) and nucleotide-binding oligomerization domain like receptors (NLRs) trigger inflammatory cytokine messenger RNA synthesis in macrophages and microglia in response to microbial components and can be also activated by endogenous damage-associated molecular pattern molecules released from necrotic brain cells^7^. Inflammasome is a kind of a tissue-damage sensor necessary for the conversion of the pro-form of IL-1β to the mature, active form and is also implicated in a form of cell death called pyroptosis^8^,^9^,^10^,^11^. IL-1β, caspase-1 and NLR family, pyrin domain containing 3 (NLRP3) have been reported to play critical roles in rodent models of ischaemic brain injury^12^,^13^,^14^ and the *IL-1β* gene single-nucleotide polymorphism is reportedly associated with stroke^15^.

Extracellular secretion of IL-1β and IL-18 requires two distinct signals: the signal driven by pattern recognition receptors, which induces the expression of pro-IL-1β and pro-IL-18 mRNAs (signal 1), and the activation of inflammasome (signal 2), which stimulates the cleavage of caspase-1 (refs 10, 16, 17). Inflammasome consists of one of the NLRP proteins NLRC4 or AIM2 along with apoptosis-associated speck-like protein containing a caspase-recruitment domain (ASC) and caspase-1. The NLRP3 inflammasome is activated by various stimuli including the rupture of lysosome membranes by phagocytosis of crystals such as monosodium urate (MSU), alum, silica and cholesterol, the disturbance of cellular ion balance by ATP and nigericin, and infection with pathogens^18^,^19^,^20^. Various protein kinases including PKR, PKC, DAPK, IRAK, Syk and JNK have been reported to be required for activation of the inflammasomes^21^,^22^,^23^,^24^,^25^,^26^. In particular, it has been demonstrated that Syk is involved in the phosphorylation of murine ASC (Tyr-146 of human ASC), forming ASC specks and activating NLRP3 and AIM2 (ref. 27). However, Syk deficiency results in only partial impairment of the NLRP3 inflammasome activation and Syk is not essential for inflammasome activation in dendritic cells (DCs)^25^,^27^. Thus, other tyrosine kinases (TKs) are probably involved in ASC phosphorylation and thus in NLPR3 inflammasome activation.

Bruton's TK (BTK), a member of the Tec family of non-receptor TKs that is structurally related to Syk, is expressed in B cells and myeloid cells, and plays critical roles not only in B-cell receptor signalling but also in TLR signalling^28^,^29^,^30^. BTK is activated by Lyn or Syk, which triggers the activation of phospholipase Cγ (PLCγ) to promote Ca^2+^ influx^31^. Dysfunctional mutations in BTK cause the failure of B-cell development, leading in humans to X-linked agammaglobulinemia (XLA), a prototypic primary humoral immunodeficiency, and in mice to the related condition X-linked immunodeficiency (Xid)^32^. Ibrutinib (PCI-32765) is a potent covalent inhibitor of BTK that was recently approved by the Food and Drug Administration for the treatment of mantle cell lymphoma and chronic lymphocytic leukemia^33^. In addition, deficiency of BTK or BTK inhibitors alleviates Th17-cell-related inflammatory responses such as elimination of *Candida albicans*, experimental autoimmune encephalomyelitis and collagen-induced arthritis^34^,^35^,^36^. Therefore, BTK could be involved not only in cancer but also in autoimmunity and inflammatory diseases^37^.

In this study, we demonstrate that BTK plays a critical role in activation of the NLRP3 inflammasome but not the AIM2 inflammasome. BTK physically interacts with NLRP3 and ASC, resulting in the induction of ASC oligomerization and caspase-1 activation in a kinase activity-dependent manner *in vitro*. We also demonstrate that BTK is activated in infiltrating macrophages/neutrophils in a brain ischaemia/reperfusion model, and that ibrutinib strongly protects against brain injury. These results may enhance our understanding of the mechanism underlying NLRP3 inflammasome activation and the effectiveness of BTK inhibitors for the treatment of acute inflammatory diseases including ischaemic stroke.

## Results

### BTK is required for the NLRP3 inflammasome activation

**Figure 1:**
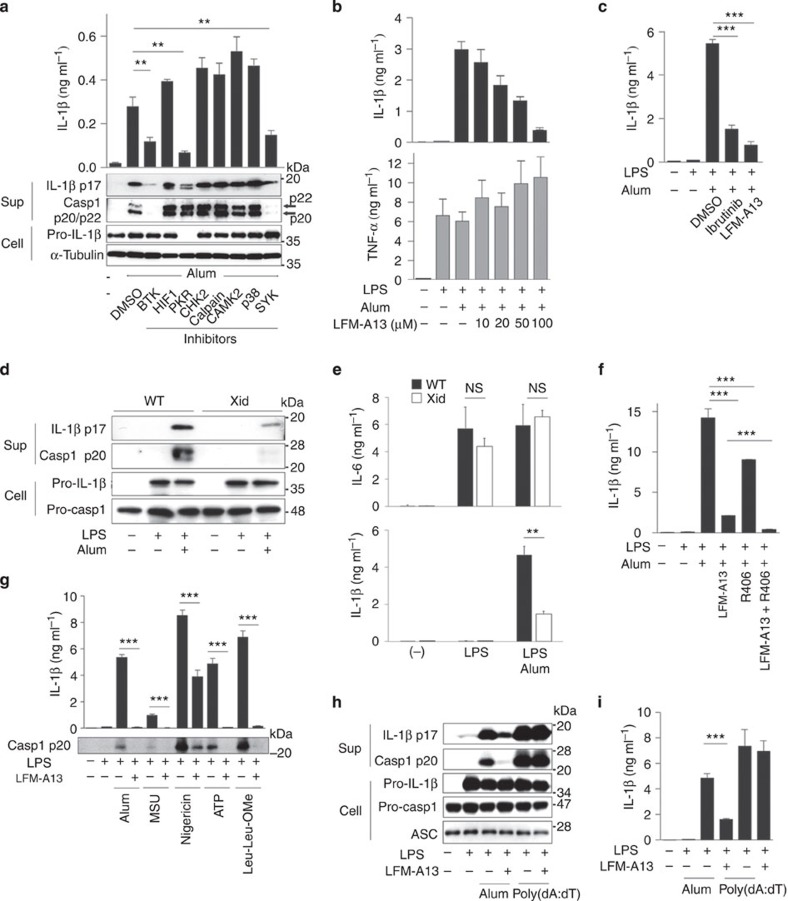
BTK inhibitors and its dysfunctional mutation suppress NLRP3 inflammasome activation. (**a**) Enzyme-linked immunosorbent assay (ELISA) of human IL-1β in supernatants and immunoblot analysis of human IL-1β p17, caspase-1 p20/p22 in supernatants and pro-IL-1β in cell lysates of THP-1-Mφs that were pretreated with the indicated inhibitors for 30 min and then stimulated with alum for 6 h. ELISA of murine IL-1β (**b**,**c**) and TNF-α (**b**) in supernatants of LPS-primed murine peritoneal macrophages that were pretreated with LFM-A13 and then stimulated with alum for 3 h. (**d**,**e**) Immunoblot analysis of the indicated proteins (**d**) and ELISA of murine IL-1β and IL-6 in supernatants of LPS-primed peritoneal macrophages from Xid and WT mice stimulated with alum for 6 h. (**f**) ELISA of murine IL-1β in supernatants of LPS-primed murine peritoneal macrophages pretreated with LFM-A13 and/or Syk inhibitor (R406), then stimulated with alum for 3 h. (**g**) ELISA of murine IL-1β and immunoblot analysis of murine caspase-1 p20 in supernatant of LPS-primed murine peritoneal macrophages stimulated with the indicated NLRP3 inflammasome activators for 3 h. Immunoblot analysis of murine IL-1β p17, caspase-1 p20 in supernatants, pro-IL-1β, pro-caspase-1 and ASC in cell lysates (**h**), and ELISA of murine IL-1β in supernatants (**i**) of LPS-primed murine peritoneal macrophages that were pretreated with LFM-A13 and then stimulated with alum or poly(dA:dT) for 3 h. Data are representative of three independent experiments. Data are presented as mean±s.d. (triplicate). **P<0.01; ***P<0.003. Two-sided Student's *t*-test.

To identify the molecules that modulate the NLRP3 inflammasome activation, we screened various types of pharmacological signal inhibitors. The human monocyte cell line THP-1 was differentiated into macrophages (THP-1-Mφs) by phorbol 12-myristate 13-acetate (PMA) priming (signal 1); this treatment induced intracellular pro-IL-1β accumulation without engaging TLR signalling pathways. Next, THP-1-Mφs were pretreated with each of the inhibitors and then stimulated with alum to activate the NLRP3 inflammasome (signal 2). Among the inhibitors, the PKR inhibitor ASN11124542, the BTK inhibitor LFM-A13 and the Syk inhibitor R406 significantly suppressed IL-1β secretion (Fig. 1a). Although PKR has already been reported to modulate inflammasome activation^21^, we found that the PKR inhibitor mainly suppressed intracellular pro-IL-1β accumulation in THP-1-Mφs (Fig. 1a). On the other hand, LFM-A13 and R406 suppressed IL-1β secretion and caspase-1 activation without affecting pro-IL-1β accumulation in THP-1-Mφ (Fig. 1a). Similarly, LFM-A13 suppressed alum-induced IL-1β secretion from murine peritoneal macrophages primed with not only lipopolysaccharide (LPS) but also poly(I:C) (Fig. 1b and Supplementary Fig. 1a). On the other hand, TNF-α secretion was not affected by LFM-A13 in this culture (Fig. 1b). Therefore, we decided to focus on BTK inhibitors. Another BTK inhibitor PCI-32765 (ibrutinib) also significantly suppressed alum-induced IL-1β secretion from peritoneal macrophages primed with LPS (Fig.1c). LPS-primed peritoneal macrophages from Xid mice, which carry a dysfunctional mutation in the pleckstrin homology domain of BTK^38^, exhibited substantially lower levels of pro-IL-1β processing and caspase-1 activation compared with those from wild-type (WT) mice (Fig. 1d,e and Supplementary Fig. 1b), whereas IL-6 secretion was not significantly impaired by BTK deficiency (Fig. 1e). Consequently, alum-induced macrophage and neutrophil infiltration into the peritoneal cavity, which has been shown to be dependent on the NLRP3 inflammasome activation^27^, was significantly lower in Xid mice than in WT mice (Supplementary Fig. 1c). Collectively, these results suggest that BTK is deeply involved in inflammasome activation for IL-1β production.

In accordance with recent reports^25^,^27^, the Syk inhibitor R406 partially inhibited IL-1β secretion from alum-stimulated macrophages and a combination of R406 and LFM-A13 suppressed IL-1β secretion more profoundly than either did alone (Fig. 1f), suggesting that Syk and BTK redundantly regulate inflammasome activation. In peritoneal macrophages, the effect of the BTK inhibitor was more profound than that of the Syk inhibitor (Fig. 1f).

### BTK is required for NLRP3 but not AIM2 inflammasome

Next, to investigate which types of inflammasome are activated through BTK, we examined various activators of signal 2. LFM-A13 suppressed IL-1β secretion and caspase-1 activation in peritoneal macrophages induced by any of the tested NLRP3 inflammasome activators, namely alum, MSU (crystals), ATP, nigericin (a potassium ionophore) and Leu-Leu-OMe (a lysosomal destabilizer) (Fig. 1g and Supplementary Fig. 1d). Poly(dA:dT) activates the AIM2 inflammasome, which is a cytosolic DNA sensor and also contains ASC^39^. Although Syk inhibition severely reduced the AIM2 inflammasome activation (Supplementary Fig. 1e and ref. 27), LFM-A13 showed little effects on poly(dA:dT)-induced caspase-1 activation, pro-IL-1β processing or mature IL-1β secretion in murine peritoneal macrophages and THP-1-Mφs (Fig. 1h,i and Supplementary Fig. 1f). Similar results were obtained in human blood monocytes (Supplementary Fig. 1f). Knockdown of *BTK* in THP-1-Mφs resulted in a significant reduction in IL-1β secretion from alum-stimulated THP-1-Mφs, but not from those stimulated with poly(dA:dT) (Supplementary Fig. 1g,h). These results suggest that BTK is involved in the NLRP3 inflammasome but not the AIM2 inflammasome activation.

### BTK promotes ASC oligomerization and redistribution

**Figure 2:**
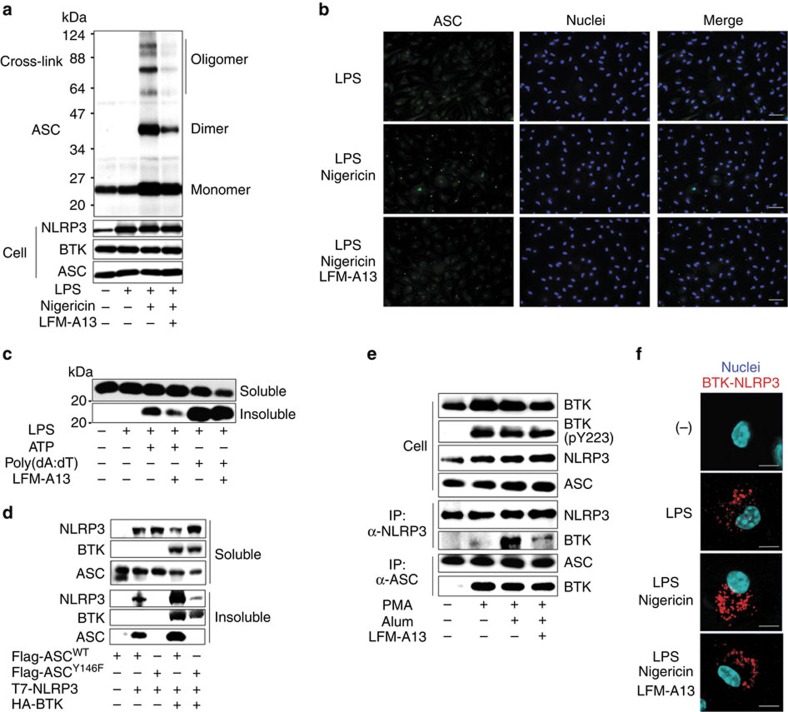
BTK promotes ASC aggregation and interacts with ASC and NLRP3. (**a**) Immunoblot analysis of ASC in BS3-treated or -untreated cell lysates of LPS-primed murine peritoneal macrophages that were pretreated with LFM-A13 and then stimulated with nigericin for 30 min. (**b**) Immunostaining of ASC of LPS-primed murine peritoneal macrophages that were pretreated with LFM-A13 and then stimulated with nigericin for 30 min. Nuclei were counterstained with Hoechst 33342. ASC, green; nuclei, blue. Scale bars, 100 μm. (**c**) Immunoblot analysis of ASC in the Triton X-soluble and -insoluble fractions of LPS-primed murine peritoneal macrophages that were pretreated with LFM-A13 and then stimulated with ATP or poly(dA:dT) for 2 h. (**d**) Immunoblot analysis of human NLRP3, BTK and ASC in the Triton X-soluble and -insoluble fractions of HEK 293T cells transfected with Flag-ASC^WT^ or -ASC^Y146F^, T7-NLRP3, or HA-BTK. Six hours after the transfection, LFM-A13 was added to the cell culture. Data are representative of three independent experiments. (**e**) Co-immunoprecipitation and immunoblot assays of HA-BTK and NLRP3 or ASC from PMA-primed and -unprimed THP-1 cells that stably expressed HA-BTK. Thirty minutes after the pretreatment with LFM-A13, cells were stimulated with alum for 3 h. (**f**) *In-situ* proximity-ligation assay of BTK–NLRP3 complexes in LPS-primed murine peritoneal macrophages pretreated with LFM-A13 and then stimulated with nigericin for 30 min. BTK–NLRP3 complexes, red; nuclei, blue. Data are representative of three independent experiments. Scale bars, 10 μm.

We next investigated the mechanism of the involvement of BTK in the NLRP3 inflammasome activation. Oligomerization and speck formation of ASC have been shown to be required for inflammasome activation^27^. We found that pretreatment with BTK inhibitor reduced ASC oligomerization and the size of ASC specks in nigericin-stimulated macrophages, but did not substantially affect the number of specks (Fig. 2a,b). In HEK293 cells, BTK overexpression enhanced NLRP3-mediated ASC oligomerization, whereas BTK inhibitor suppressed ASC oligomerization (Supplementary Fig. 2a). ASC has been shown to be redistributed to the Triton X-insoluble fraction by stimulation with inflammasome activators^27^. BTK inhibition substantially reduced ASC redistribution into the Triton X-insoluble fraction in macrophages stimulated by ATP but not by poly(dA:dT) (Fig. 2c). Similar results were obtained in HEK293 cells overexpressing NLRP3, ASC and BTK (Supplementary Fig. 2b). Hara *et al.*^27^ have recently reported that tyrosine phosphorylation of human ASC-Y144 (corresponding to Y146 in mouse ASC) is required for inflammasome activation, and that phosphorylated ASC can be detected in the Triton X-insoluble fraction. Consistent with this report, NLRP3 overexpression induced redistribution of WT ASC but not ASC^Y146F^, which was strongly enhanced by BTK overexpression (Fig. 2d). BTK kinase activity is more likely to be required for ASC phosphorylation, as redistribution of ASC due to BTK overexpression was severely reduced in the presence of the BTK inhibitor (Supplementary Fig. 2b). Moreover, ASC redistribution was hardly observed when TK domain-deleted BTK (BTKΔTK) (Supplementary Fig. 2b) or kinase-dead mutant BTK (R525Q or E567K) (Supplementary Fig. 3) were overexpressed. Collectively, these results suggest that BTK activity promotes ASC oligomerization and redistribution in macrophages.

### BTK interacts with NLRP3 and ASC

We next examined the mechanism of BTK-mediated ASC oligomerization. BTK is known to phosphorylate PLCγ to induce Ca^2+^ influx, one of the triggers of the NLRP3 inflammasome activation^18^. We were unable, however, to observe any suppressive effects of LFM-A13 on the Ca^2+^ influx in THP-1 cells (Supplementary Fig. 4) and on phosphorylation of PLCγ1 and PLCγ2 (Supplementary Fig. 5), suggesting that BTK is not involved upstream of NLRP3. BTK seems to inhibit NLRP3 inflammasome activation independent of effects on phagocytosis or priming, because BTK inhibitor suppressed IL-1β maturation and caspase-1 activation induced by nigericin and ATP, which are independent on phagocytosis (Fig. 1g and Supplementary Fig. 1d). Furthermore, BTK inhibitor also suppressed caspase-1 activation induced by a lysosome rupture agent, Leu-Leu-OMe (Fig. 1g), suggesting that BTK acts after phagocytosis and lysosome rupture. A previous report suggested a partial reduction of mitocondorial reactive oxygen species in macrophages from Xid mice^34^. We confirmed this using the BTK inhibitor (Supplementary Fig. 6), indicating that although BTK has a unique capacity to activate NLRP3 inflammasome directly through a physical interaction, BTK may also be involved in inflammasome activation through indirect mechanisms such as reactive oxygen species generation.

Next, we investigated the possibility that BTK interacts with the constituents of the NLRP3 inflammasome. For this purpose, we generated THP-1 cells stably expressing BTK (BTK-THP-1 cells), which were differentiated into BTK-THP-1-Mφs by PMA treatment. Higher levels of caspase-1 activation and mature IL-1β secretion in response to alum were observed in BTK-THP-1-Mφs compared with parental THP-1-Mφs (Supplementary Fig. 7a). Importantly, BTK overexpression induced to activate caspase-1 without increasing pro-caspace-1 expression, even though the cells were not stimulated with alum (Supplementary Fig. 7a). IL-1β secretion was still suppressed by BTK inhibitor treatment (Supplementary Fig. 7b). Immunoprecipitation and western blotting analysis revealed that PMA treatment activated BTK by phosphorylating its Tyr-223 and induced BTK to interact with both ASC and NLRP3 (Fig. 2e). The interaction of BTK with NLRP3 was weak in BTK-THP-1-Mφs, but was substantially strengthened by stimulation with alum (Fig. 2e). To confirm these findings, we investigated the BTK–NLRP3 interaction in murine peritoneal macrophages using *in-situ* proximity-ligation assay. We found that LPS priming (signal 1) induced BTK–NLRP3 interaction, and that activating the NLRP3 inflammasome with nigericin (signal 2) increased this interaction, mainly at the perinuclear region, whereas LFM-A13 abrogated it (Fig. 2f). By administering various deletion mutants of BTK, NLRP3 and ASC, we found that the ASC-pyrin domain bound to the BTK-TK domain, whereas the NLRP3-NACHT and -leucine-rich repeat domains bound to both the BTK-SH2/3 and -TK domains (Supplementary Fig. 8). These results suggest that BTK functions as a platform protein for the interaction between NLRP3 and ASC. We propose that first BTK activated by signal-1 physically interacts with ASC, then signal-2 induces recruitment of NLRP3 to this BTK–ASC complex, further inducing ASC oligomerization.

### BTK inhibitor has a neuroprotective effect on brain ischaemia

**Figure 3:**
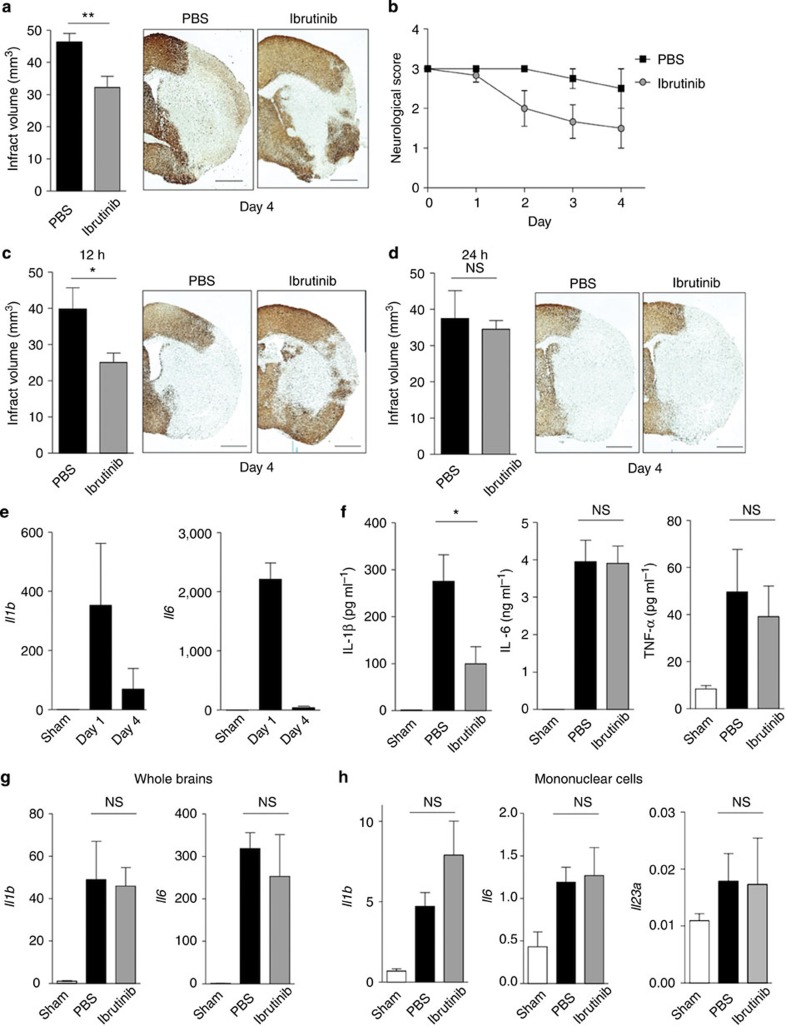
Neuronal protection and inhibition of IL-1β maturation by ibrutinib in ischaemic brain injury. Infarct volume on day 4 after stroke onset (**a**) and neurological scores (**b**) of mice treated with PBS or ibrutinib (3.125 mg kg^−1^ per day on Day 0, 1) immediately after stroke onset (*n*=9 for PBS and *n*=8 for ibrutinib). Infarct volume on day 4 after stroke onset of mice treated with PBS or ibrutinib 12 (**c**) or 24 h (**d**) after stroke onset (*n*=6). Scale bars, 1 mm (**a**,**c**,**d**). (**e**) mRNA levels of IL-1β or IL-6 in the ischaemic brain tissue on day 1 and 4 after stroke onset (*n*=3). (**f**) Enzyme-linked immunosorbent assay of IL-1β, IL-6 or TNF-α in the ischaemic brain lysate on day 1 after stroke onset (*n*=3). (**g**) mRNA levels of IL-1β or IL-6 in the ischaemic brain on day 1 after stroke onset (*n*=3). (**h**) mRNA levels of IL-1β, IL-6 or IL-23 in mononuclear cell fractions on day 1 after stroke onset (*n*=5). Data are representative of three independent experiments. Data are presented as mean±s.e.m. *P<0.05; **P<0.01. Two-sided Student's *t*-test.

NLRP3-mediated IL-1α/β has been shown to contribute to the progression of cerebral ischaemic injury^14^. We confirmed that IL-1α/β knockout mice, in which both IL-1α and IL-1β were functionally deficient^40^, were highly resistant to ischaemic brain injury (Supplementary Fig. 9a). Previous studies have shown that caspase-1 deficiency and caspase-1 inhibitor strongly suppress ischaemic brain damage^41^,^42^. Thus, the inflammasome-caspase-1 cascade plays an important role in brain damage. Therefore, we investigated the therapeutic effect of ibrutinib on the stroke model. The dosage of ibrutinib used in this experiment was equal to that used in a previously published mouse CIA model^36^,^43^. When ibrutinib was administered twice by intravenous injection (3.125 mg per kg body weight), once immediately after reperfusion (on day 0) and once 24 h after later (on day 1), the infarct area was significantly reduced compared with PBS-injected control (Fig. 3a). Neurological deficits were also improved by ibrutinib treatment (Fig. 3b). To determine the therapeutic time window, we examined the effect of BTK inhibitor administration 12 and 24 h after stroke onset. Administration of ibrutinib 12 h after stroke onset still significantly reduced ischaemic brain damage (Fig. 3c); administration 24 h after stroke onset, however, did not result in any protective effect (Fig. 3d). This indicates that ibrutinib inhibits early events occurring within 24 h after ischaemia.

Next, to investigate the effect of ibrutinib on inflammation after ischaemic brain injury, inflammatory cytokine expression was measured. The mRNA levels of IL-1β and IL-6 in infarct-side brain tissue were increased on day 1 and decreased on day 4 after stroke onset (Fig. 3e). When mice were treated with ibrutinib or PBS on day 0, mature IL-1β protein levels on day 1 after stroke onset were much lower in the brain lysates of ibrutinib-treated mice than in control mice, whereas the IL-6 and TNF-α protein levels were similar (Fig. 3f). The mRNA levels of IL-1β, IL-6 and IL-23 were not significantly different between ibrutinib- and PBS-treated mice in the infarct side brain or in the infiltrating mononuclear cells (Fig. 3g,h). These results are consistent with the observation that BTK is involved in inflammasome activation (signal 2) but not signal 1 activation *in vitro* (Fig. 1). Therefore, ibrutinib could ameliorate ischaemic brain injury by inhibiting caspase-1 activation and/or IL-1β maturation. This hypothesis was supported by our finding that ibrutinib showed no further protective effect in the stroke model of IL-1-deficient mice (Supplementary Fig. 9b,c).

### BTK inhibitor suppresses inflammasome in the ischaemic brain

**Figure 4:**
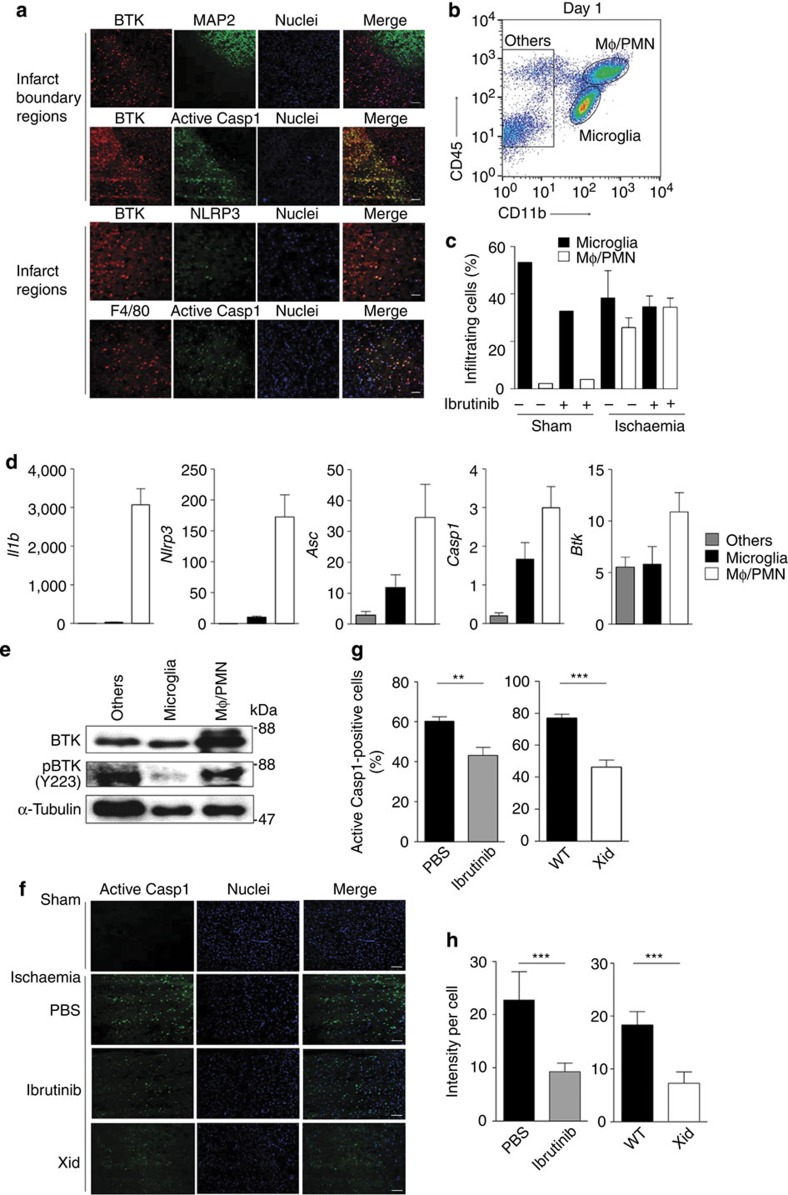
Activation of BTK and caspase-1 in infiltrating macrophages. (**a**) Immunofluorescence staining for BTK and MAP2, BTK and active caspase-1 in the infarct boundary regions, BTK and NLRP3, and F4/80 and active caspase-1 in the infarct regions. Brain specimens were harvested on day 1 after stroke onset. (scale bars, 50 μm). (**b**) Macrophages/polymorphonuclear neutrophiles (Mφ/PMN), microglia and other cell fractions were sorted by FACS from a pool of mononuclear cells separated from the brain on day 1 after stroke onset. Mφ/PMN fraction: CD45 high, CD11b high; microglia fraction: CD45 intermediate, CD11b intermediate; and other cell fractions: CD11b negative. (**c**) The numbers of cerebral infiltrating Mφ/PMNPMN or microglia on day 1 (*n*=1 for sham and *n*=4 for ischaemia). (**d**,**e**) mRNA levels of inflammasome components and BTK protein levels in microglia, Mφ/PMN and other cell factions on day 1 after stroke onset. *n*=8 for (**d**) and representative data from three independent experiments (**e**). (**f**) Staining for active caspase-1 in the infarct area on day 1 after stroke onset (scale bars, 50 μm). (**g**) The numbers of active caspase-1-positive cells in the infarct area on day 1 after stroke onset (*n*=6). (**h**) Relative fluorescence intensities of active caspase-1 in the cells of the infarct area on day 1 after stroke onset (*n*=15). Data are representative of three independent experiments. Data are presented as mean±s.e.m. *P<0.05; **P<0.01; ***P<0.001. Two-sided Student's *t*-test.

We investigated the role of BTK in inflammasome activation induced by ischaemic brain injury. The expression of BTK and inflammasome in the ischaemic brain was measured on day 1 after stroke onset. BTK-positive cells were more abundant in the MAP2-negative infarct region, where they were co-stained with active caspase-1, NLRP3 and F4/80 (Fig. 4a), suggesting that BTK and inflammasome are activated in infiltrating macrophages or microglia in the infarct area. To investigate whether ibrutinib affected infiltration of inflammatory cells into the infarct areas, microglia, macrophages and other cells were isolated by fluorescence-activated cell sorting (FACS) (Fig. 4b). To determine which types of cells express mature IL-1β, microglia, macrophages and other cells were isolated by FACS (Fig. 4b). CD45^intermediate^CD11b^intermediate^ population represented microglia fraction, and CD45^high^CD11^high^ fraction, which we called ‘macrophages/neutrophils (Mφ/PMN)' fraction, appeared after ischaemic brain injury and ∼20%–50% of this fraction contained Gr1^−^ macrophages and Gr1^+^ neutrophils on day 1 after stroke onset (Fig. 4c and Supplementary Fig. 10). As neutrophils also have been reported to express NLRP3, ASC and caspase-1, and produce mature IL-1β^44^, inflammasome may be activated in both macrophages and neutrophils. Ibrutinib administration did not affect the population of macrophage and microglia fractions (Fig. 4c), although it slightly reduced the number of macrophages (Supplementary Fig. 10), which may reflect the infarct volume reduction by ibrutinib treatment.

As shown in Fig. 4d, macrophages/neutrophils in the CD45^high^CD11b^high^ fraction, but not microglia and other cells, expressed IL-1β and NLRP3 mRNAs. ASC and caspase-1 were expressed in both cells in microglia and macrophage fractions, although their expression levels were higher in macrophages than in microglia (Fig. 4d). Activation of BTK was observed in the macrophage/neutrophil fraction and in the other cell fraction, but not in the microglia fraction (Fig. 4e). Taking together with immunofluorescence imaging of Fig. 4a, these data suggest that BTK-mediated inflammasome activation occurred mostly in infiltrating macrophages/neutrophils.

Next, we confirmed suppression of inflammasome activation by BTK inhibition *in vivo*. Being consistent with reduced mature IL-1β production (Fig.3f), immunofluorescence staining revealed that ibrutinib treatment strongly suppressed caspase-1 activation in the infarcted area of the brain (Fig. 4f,g,h). Similarly, caspase-1 activation was significantly lower in the brain of BTK-deficient Xid mice than in that of WT mice (Fig. 4f,g,h). These results suggest that BTK inhibition suppresses inflammasome activation and therefore reduces caspase-1 activation and IL-1β secretion, thus promoting neural protection in the injured brain.

## Discussion

Recently, multiple kinases such as PKC-δ and PKR have been identified as regulators of inflammasome activation^21^,^22^ and we have shown that the TAK1-JNK pathway is necessary for ASC oligomerization induced by lysosome rupture^26^. More recently, Hare *et al.*^27^ demonstrated that Syk TK induces phosphorylation of Tyr-144 of ASC on activation of the NLRP3 and AIM2 inflammasomes, which is necessary for ASC oligomerization. However, it is still unknown whether Syk directly phosphorylates ASC or other TK(s) are involved^45^. In this study, we found that BTK, a TK structurally related to Syk, regulated activation of the NLRP3 inflammasome by interacting with NLRP3 and ASC. BTK and Syk seem to be redundant, although the role of BTK is more profound than that of Syk for the NLRP3 inflammasome activation in peritoneal macrophages. We have not strictly proven that BTK directly phosphorylates ASC, but we consider this probable, because BTK binds to ASC through its kinase domain and because BTK overexpression directly induces oligomerization of ASC in HEK293 cells in a Tyr-146-dependent manner. Further biochemical analysis is underway to demonstrate a direct tyrosine phosphorylation of ASC by BTK. At present, it is still not clear why BTK is not involved in AIM2 inflammasome activation. As Syk inhibitor but not BTK inhibitor severely suppressed poly(dA:dT)-mediated IL-1β production (Supplementary Fig. 1e and ref. 27), Syk, but not BTK, could play a dominant role for the AIM2 inflammasome activation.

BTK is a well-investigated kinase involved in the TLR signalling pathways, which are the priming step in inflammasome activation (signal 1)^10^,^17^. In spite of the previous extensive studies on Xid mice and human XLA patients, however, our study revealed that the effects of BTK inhibition on the production of proinflammatory cytokines such as IL-6 and TNF-α from macrophages were limited (Fig. 1e). On the other hand, we found that the BTK inhibitors LFM-A13 and ibrutinib, Xid mutation, or *BTK* knockdown inhibited mature IL-1β secretion from macrophages and DCs stimulated with the NLRP3 inflammasome activators. Biochemical analysis strongly suggests that BTK mostly regulates a step in the NLRP3 inflammasome activation (signal 2). Our experiments using THP-1 cells and HEK293 cells revealed that BTK also functions as a platform or scaffold protein for NLRP3 and ASC. On receiving an NLRP signal, BTK may induce the phosphorylation and oligomerization of ASC, which results in caspase-1 activation. Further study is necessary to clarify the precise mechanism of BTK-mediated caspase-1 activation.

Production of pro-inflammatory cytokines is an essential step in the progression of brain ischaemia–reperfusion injury^7^. Among the cytokines we examined, IL-1β and IL-23 are the most critical. Infarct volumes of IL-23- or IL-1β-deficient mice were up to 50% smaller than those of WT mice in the ischaemia/reperfusion model (Supplementary Fig. 9a and ref. 4). Both cytokines activate the secretion of IL-17 by γδT cells, which is also shown to promote brain injury when induced by ischaemia or experimental autoimmune encephalomyelitis^46^,^47^. As IL-23 and IL-1β have additional distinct functions, however, the precise effects of these cytokines on brain cells remain to be clarified. In addition, the inflammasome activation mechanism upstream of BTK is also unclear. Various damage-associated molecular pattern molecules including peroxiredoxin have been reported to activate TLRs (signal-1), thereby inducing inflammatory cytokines from infiltrating macrophages^48^. Although various candidates such as K^+^ ions and ATP have been proposed, the mechanism generating signal-2 in the inflammasome activation process in the damaged brain after ischaemic stroke remains to be investigated.

The role of inflammasomes, caspase-1 and their activated products IL-1α and IL-1β in ischaemic brain damage has been previously reported^12^,^13^,^14^,^41^. It has been shown that BTK expression is increased after ischaemic brain injury^49^. Along with these reports, our study strongly suggests that BTK is involved in caspase-1 activation and mature IL-1β production in post-ischaemic inflammation. As the BTK inhibitor ibrutinib has been approved for mantle cell lymphoma and chronic lymphocytic leukemia, and has been shown to be well tolerated by patients with few adverse effects, we thought that ibrutinib could also be effective against inflammasome-dependent diseases including ischaemic stroke. As expected, ibrutinib was found to be effective in our ischaemic brain injury model in mice. Although no therapeutic effect of ibrutinib was observed 24 h after stroke onset, its therapeutic time window appears to be longer than that of tPA treatment. Ibrutinib has sufficient potential to be used in a clinical trial of ischaemic stroke patients.

We noticed that IL-1β expression in the ischaemic brain of Xid (*Btk*-deficient) mice was reduced compared with WT mice (Supplementary Fig. 11a); however, this reduction was not statistically significant and not so evident compared with ibrutinib-treated mice (Fig. 3f). This may be due to more severe brain injury after ischaemia/reperfusion in Xid mice than that in WT mice because of the lack of IL-10-producing regulatory B cells in Xid mice (Supplementary Fig. 11b)^50^. In contrast, ibrutinib did not show protective effect on the stroke model of Xid mice (Supplementary Fig. 11c). These data support our proposal that ibrutinib reduces infarct volume mainly by inhibiting BTK. However, we could not completely rule out the possibility that ibrutinib may be protective by blocking other inflammatory pathways. Protective role of B cells in inflammation also suggests a limitation of the application of BTK inhibitors for inflammatory diseases; however, if BTK inhibitors are used only during the acute phase of stroke, effects on B cells may be negligible.

The NLRP3 inflammasome has been shown to be involved in the pathophysiology of various inflammatory diseases, including gout, type 2 diabetes, obesity-induced insulin resistance, atherosclerosis and Muckle–Wells syndrome^11^,^51^. Furthermore, inflammasome is also reported to be involved in neurological diseases such as Alzheimer's disease and Parkinson's disease^52^. Administration of anti-IL-1β antibody has been demonstrated to be effective in treating some of these disorders^51^,^53^. BTK inhibitors have been shown to have beneficial effects on rheumatoid arthritis and systemic lupus erythematosus in animal models^43^,^54^. Therefore, BTK inhibitors may be effective against a broad spectrum of inflammatory disorders in which the NLRP3 inflammasome is involved. Further investigation into the mechanism of BTK-mediated NLRP3 inflammasome activation will provide a new strategy for treating inflammatory diseases.

## Methods

### Mice

C57BL/6J mice were purchased from Tokyo Laboratory Animals Science Co., Ltd, CBA/N (Xid) mice were from Sankyo Labo Service Co. and CBA/J (WT control) mice were from Oriental Yeast Co., Ltd. C57BL/6 IL-1β^−/−^ mice (also deficient for IL-1α) were kindly provided by Dr Iwakura (Tokyo Science University)^55^. Male mice were used at 8–10 weeks of age. Animal experiments were performed in strict accordance with the recommendations in the Guidelines for Proper Conduct of the Animal Experiments of Science Council of Japan. All experiments were approved by the Institutional Animal Research Committee and Ethics Committee of Keio University.

### Cells

Murine peritoneal macrophages were prepared by intraperitoneal injection of thioglycollate. The peritoneal cavity was infused with 5–10 ml PBS at 5 days after injection. The fluid-filled cavity was gently shaken and fluid was withdrawn^19^. Bone marrow-derived DCs (BMDCs) were prepared from bone marrow suspensions from the femurs and tibias in mice as described^56^. Bone marrow cells were cultured in 10 ng ml^−1^ mouse granulocyte–macrophage colony-stimulating factor (PeproTech) and the 10-day-cultured BMDCs were used for the experiment. THP-1 cells (RIKEN) were cultured in RPMI 1640 medium (Invitrogen) supplemented with 10% fetal bovine serum (FBS), 2-ME (Invitrogen), L-glutamine and penicillin/streptomycin (Nacalai). HEK293 cells (RIKEN) were maintained in DMEM supplemented with 10% FBS, L-glutamine and penicillin/streptomycin. To prepare human blood monocytes, human peripheral blood mononuclear cells were isolated from heparinized whole blood using Ficoll-Paque PLUS (GE Healthcare) and then adhered on plastic flasks (Iwaki) for 3 h.

### Reagents and antibodies

LPS, nigericin, MSU, poly(I:C), poly(dA:dT) (all from Invivogen), alum (Thermo Scientific), ATP and Leu-Leu-OMe (both from Sigma-Aldrich) were used for cell stimulation. Anti-ASC (N-15) (1:1,000; Santa Cruz), anti-NLRP3 (1:1,000; Cryo-2, AdipoGen), anti-mouse IL-1β (1:500; AF-401-NA for pro-IL-1β) and anti-human IL-1β (1:500; AF-201-NA for pro-IL-1β) (both from R&D), anti-mouse IL-1β (1:125; BioVision 5129-100 for IL-1β p17), anti-mouse caspase-1 (1:500; 5B10, eBioscience), anti-human IL-1β (1:500; 2,022 for IL-1β p17), anti-human caspase-1 (1:500; 2,225 for pro-caspase-1 and 4199 for caspase-1 p20) and anti-phospho-tyrosine (1:1,000; 9,411 all from Cell Signaling), anti-BTK (NT) (1:200; 54,190, Anaspec), anti-BTK (pY223) (1:5,000; EP420Y, Epitomics), anti-HA (1:2,000; ab-hatag, Invivogen), anti-T7 (1:5,000; PM022, MBL), anti-Flag (1:2,000; M2, F3165, Sigma), anti-α-tubulin (1:2,000; DM1A, Sigma), anti-F4/80 (1:100; MCA497, Bio-Rad), anti-NeuN (1:1,000; A60, Millipore) and anti-MAP2 (1:1,000; HM-2, Sigma) were used for immunoprecipitation, western blotting, immunofluorescence staining, or *in-situ* proximity-ligation assay. FAM-FLICA *in vitro* Caspase Detection Kit (ImmunoChemistry) was used for active caspase detection. Secondary antibodies were peroxidase-conjugated AffiniPure goat anti-rabbit IgG (H+L), anti-mouse IgG (H+L), anti-rat IgG (H+L) and AffiniPure donkey anti-goat IgG (H+L), (1:5,000; all from Jackson Immunoresearch), and Alexa Fluor 488-conjugated goat anti-rabbit IgG (H+L), Alexa Fluor 488-conjugated goat anti-mouse IgG (H+L), Alexa Fluor 546-conjugated goat anti-rabbit IgG (H+L) and Alexa Fluor 546-conjugated goat anti-rat IgG (H+L) (1:300; all from Invitrogen). Pairs of biotin-labelled and -unlabelled anti-human IL-1β monoclonal Abs (mAbs) (BioLegend), anti-murine IL-1β, IL-6 and TNF-α mAbs (all from eBioscience) were used for enzyme-linked immunosorbent assay. Allophycocyanin-conjugated anti-mouse Gr1 mAb (RB6-8C5), fluorescein isothiocyanate (FITC)- or phycoerythrin-conjugated anti-mouse F4/80 mAb (BM8), allophycocyanin- or PerCP conjugated anti-mouse CD11b mAb (M1/70), or FITC-conjugated anti-mouse CD45 mAb (30-F11) (1:400; all from eBioscience) were used for flow cytometric analysis. Pharmacological inhibitors are listed in Supplementary Table 1.

### THP-1 cells stably expressing BTK

BTK complementary DNA expression lentivirus vectors were transduced into HEK293 cells along with pCMV-VSV-G-RSV-Rev (RIKEN) and pMDLg/p-RRE as described^57^. Eighteen hours after transduction, the vector-containing culture medium was replaced with fresh culture medium; 48 h later, the lentivirus-containing medium was collected, passed through a 0.45-μm filter and concentrated through centrifugation (8,400*g* at 4 °C for 16 h). The lentivirus pellets were resuspended in PBS. THP-1 cells were seeded on 24-well plates at 5 × 10^4^ cells per well and the lentivirus suspensions were then added into the wells. Ten and 16 days after the transfection, Venus-highly expressing cells were sorted using a FACSAria II (BD).

### THP-1 cells with *BTK* knockdown

The human BTK short hairpin RNA (shRNA) expression vector (40 μg) was transduced into THP-1 cells (3 × 10^7^ cells) by electroporation using a Gene Pulser (Bio-Rad). Cells were then cultured in RPMI 1,640 medium supplemented with 10% FBS, 2-mercaptoethanol, L-glutamine, penicillin/streptomycin and 2 mg ml^−1^ G418 (Nacalai). A non-targeting shRNA vector was used as a control.

### Plasmids

Flag-tagged human ASC, T7-tagged human NLRP3 and HA-tagged human BTK were constructed into the pCMV expression vector. After construction, the point mutations and deletion mutations in the gene encoding human BTK, ASC or NLRP3 were introduced into each expression vector by site-directed mutagenesis. Human WT-BTK and XLA-type BTK mutations were cloned into the lentivirus vector CSII-EF-IRES2-Venus (RIKEN). Human BTK shRNA (target region, 5′-AAACAGTGATCTGGTTCAGAAA-3′) was cloned into psiRNA-h7SKneo (Invivogen)^58^.

### Quantitative real-time PCR

mRNA from the ischaemic brain tissues or infiltrating inflammatory cells prepared by Percoll gradient centrifugation were extracted using RNAiso (TaKaRa) or ReliaPrep RNA Cell Miniprep System (Promega), respectively. Real-time PCR was performed on cDNA samples using EverGreen. The relative quantitation value is expressed as 2-ΔCt, where ΔCt is the difference between the mean Ct value of duplicate measurements of the sample and the endogenous hypoxanthine phosphoribosyltransferase 1 control. Primer sequences were as follows: mouse il-1b forward 5′-CAGGCAGGCAGTATCACTCA-3′ and reverse 5′-AGGCCACAGGTATTTTGTCG-3′; mouse il-6 forward 5′-ATGGATGCTACCAAACTGGAT-3′ and reverse 5′-TGAAGGACTCTGGCTTTGTCT-3′; mouse il-23 forward 5′-AGCGGGACATATGAATCTACTAAGAGA-3′ and reverse 5′-GTCCTAGTAGGGAGGTGTGAAGTTG-3′; mouse nlrp3 forward 5′-ATGCTGCTTCGACATCTCCT-3′ and reverse 5′-AACCAATGCGAGATCCTGAC-3′; mouse asc forward 5′-GAAGCTGCTGACAGTGCAAC-3′ and reverse 5′-GCCACAGCTCCAGACTCTTC-3′; mouse caspase-1 forward 5′-AGATGGCACATTTCCAGGAC-3′ and reverse 5′-GATCCTCCAGCAGCAACTTC-3′; mouse btk forward 5′-GGGTCTCTGAGTGAGCCTTC-3′ and reverse 5′-GTTTGTGTACTAAAGGACTGTGGG-3′.

### Stimulation with inflammasome activators

Cells were plated at a density of 8 × 10^5^ cells per well in 24-well plates. Culture medium was replaced with Opti-MEM (Invitrogen) before stimulation. Human monocytes, murine macrophages and murine BMDCs were primed with 50 ng ml^−1^ LPS or poly(I:C) for 3 h and then stimulated with alum (400 μg ml^−1^), MSU (400 μg ml^−1^), nigericin (1.2 μM), ATP (2 mM) or Leu-Leu-OMe (1 mM) for 3 h. Poly(dA:dT) (2.6 μg ml^−1^) was introduced into primed macrophages using Lipofectamine LTX (Invitrogen). THP-1 cells were primed with PMA (0.5 μM) for 3 h and plated at a density of 8 × 10^5^ cells per well in 24-well plates. On the next day, THP-1-derived macrophages (THP-1-Mφs) were stimulated with alum (400 μg ml^−1^) or poly(dA:dT) (2.6 μg ml^−1^) for 3 h. Various inhibitors (Supplementary Table 1) or dimethylsulfoxide were added to cell cultures 30 min before induction of the inflammasome activation.

### Reconstruction of the inflammasome system into HEK293 cells

HEK293 cells (5 × 10^5^ cells) were plated in six-well plates and the next day transfected with Flag-ASC^WT^ or Flag-ASC^Y146F^ (100 ng), T7-NLRP3 (250 ng), or HA-BTK or HA-BTKΔTK (250 ng). At 6 h after the transfection, LFM-A13 was added to the cell culture. Twenty-four hours after transfection, the cells were lysed and analysed by western blotting.

### Cross-linking of ASC oligomers

Murine peritoneal macrophages stimulated with nigericin (1 μM) for 30 min or HEK293 cells transfected with the plasmids expressing Flag-NLRP3, Flag-ASC and/or HA-BTK were lysed with a lysis buffer (20 mM Hepes-KOH (pH 7.5), 150 mM KCl, 1% NP-40, 0.1 mM phenylmethylsulfonyl fluoride and protease inhibitor cocktail (Nacalai Tesque))^39^ and centrifuged at 6,000 r.p.m. for 10 min, and the pellets were resuspended in CHAPS buffer (20 mM Hepes–KOH (pH 7.5), 5 mM MgCl_2_, 0.5 mM EGTA and 0.1% CHAPS)^59^. The resuspended pellets were cross-linked for 30 min at room temperature with a BS3 cross-linker (4 mM) (Thermo Scientific) and dissolved in SDS sample buffer.

### Immunoprecipitation

THP-1-Mφs, THP-1 or HEK293 cells transfected with the plasmids expressing Flag-NLRP3, Flag-ASC or HA-BTK were lysed with cell lysis buffer (50 mM Tris (pH 8.0), 280 mM NaCl, 0.5% NP-40, 0.2 mM EDTA, 2 mM EGTA, 10% glycerol, 1 mM dithiothreitol and protease inhibitor cocktail) and centrifuged at 15,000 r.p.m. at 4 °C for 5 min. The supernatants were incubated with Protein A/G-Sepharose (GE Healthcare) and anti-NLRP3 mAb, anti-ASC polyclonal Ab (pAb) or anti-Flag mAb, depending on the target protein. The polypeptides in the precipitated complexes were analysed by western blotting.

### Western blot analysis

Cells were lysed with the same cell lysis buffer used for immunoprecipitation and then boiled in SDS sample buffer. Cell culture supernatants were concentrated with methanol/chloroform and the pellets were lysed in the SDS sample buffer. Triton X-100-soluble and -insoluble fractions were prepared as described previously^27^. Briefly, cells were lysed with the cell lysis buffer (50 mM Tris-HCl (pH 7.6), 0.5% Triton X-100, protease inhibitor cocktail and phosphatase inhibitor cocktail). The lysates were centrifuged at 6,000*g* at 4 °C for 15 min and the pellets and supernatants were used as the Triton X-insoluble and Triton X-soluble fractions, respectively. Images have been cropped for presentation. Full-length images of western blottings in the main figures are shown in Supplementary Fig. 12.

### Mouse focal brain ischaemia model

Male mice, aged 8–12 weeks and weighing 20−30 g, were used for focal brain ischaemia experiments^4^,^48^. There was no significant difference in weight or age between WT mice and any of the knockout groups. We used a transient middle cerebral artery occlusion (MCAO) model induced by means of an intraluminal suture. The method of inducing this transient suture MCAO model has been described previously^4^. A >60% reduction in cerebral blood flow was confirmed by laser Doppler flowmetry (Supplementary Table 2) and head temperature was kept at 36 °C using a heat lamp. Sixty minutes after MCAO, the brain was reperfused by withdrawal of the intraluminal suture. Neurological function was evaluated using a previously described four-point scale neurological score method (0=no observable deficit, 1=forelimb flexion, 2=decreased resistance to lateral push without circling, 3=same behaviour as grade 2, with circling) and assessed in a blinded manner^60^. For measurement of the infarct volume, 1-mm-thick serial coronal slices from the brains were embedded in paraffin sections and immunostained with MAP2-specific antibody as described^4^.

### Ibrutinib administration

PCI-32765 (Ibrutinib) (Biochempartner) was dissolved in dimethylsulfoxide and diluted with PBS (3.125 mg per kg body weight)^36^,^43^ and sonicated using Biorupture (Cosmo Bio). After the solution was emulsified with 1% corn oil (Sigma) and filtrated through a 0.22-μm filter, it was administered intravenously.

### Preparation of inflammatory cells from infarcted brain

Methods of preparation of inflammatory cells have been described previously^4^. At each time point, mice were transcardially perfused with ice-cold PBS to exclude blood cells. The forebrain was dissected from the cerebellum and suspended in RPMI-1640 medium. The suspension was digested with type IV collagenase (1 mg ml^−1^, Sigma-Aldrich) and DNase I (50 μg ml^−1^, Roche) at 37 °C for 45 min in a shaker at 180 times per min. Mononuclear cells were isolated by 37%–70% Percoll (GE Healthcare) density gradient centrifugation. Inflammatory cells were removed from the interface and washed with RPMI-1640 for further analysis.

### Adoptive transfer of B cells

CD19^+^ B cells were prepared from the spleen and lymph nodes of CBA/J mice by positive selection using magnetic beads. Xid (CBA/N) mice were administered CD19^+^ B cells (5 × 10^6^) into the tail vein immediately after ischaemia/reperfusion.

### Immunofluorescence staining

LPS-primed peritoneal macrophages were seeded on coverslips, stimulated with nigericin (1 μM) for 30 min and then fixed and permeabilized in 2.5% formaldehyde and 0.5% Triton X-100. The cells were incubated with anti-ASC pAb followed by Alexa Fluor 488-conjugated anti-rabbit IgG pAb. Nuclei were counterstained with Hoechst 33342. The cells were examined under a fluorescence microscope (Keyence). Coronal slices from ischaemic brain were embedded in Tissue-Tec O.C.T. Compound (Sakura Finetechnical Co., Ltd) and flash frozen in liquid nitrogen and stored at −80 °C. Sections of 8 μm in thickness were fixed for 15 s with acetone, then rinsed with PBS. Blocking with Blocking One Histo (Nacalai Tesque) was applied for 1 h at room temperature. Section were washed with PBS and then incubated with anti-BTK pAb, anti-F4/80 mAb, anti-NeuN mAb, anti-MAP2 mAb or anti-NLRP3 mAb followed by Alexa Fluor 488- or 546-conjugated goat anti-rabbit, mouse or rat IgG (H+L) or incubated with FLICA.

### *In-situ* proximity-ligation assay

LPS-primed or -unprimed peritoneal macrophages were seeded on coverslips, stimulated with nigericin (1 μM) for 30 min and then fixed and permeabilized in 2.5% formaldehyde and 0.5% Triton X-100. The cells were incubated for 1 h at 37 °C with anti-BTK and anti-NLRP3 Abs. The cells were washed and allowed to react to a pair of Duolink proximity probes (Olink Bioscience) as described previously^61^. Nuclei were counterstained with Hoechst 33342. Confocal image acquisition was performed using a Zeiss LSM 710 laser scanning microscope (Carl Zeiss).

### Peritonitis

CBA/J (WT) or CBA/N (Xid) mice were challenged intraperitoneally with 0.5 ml alum (200 μg) or PBS. The mice were killed 15 h after injection of alum and peritoneal cavities were lavaged with 6 ml PBS. Live peritoneal cells were counted according to the trypan blue dye-exclusion method and then allowed to react to anti-mouse CD16 mAb. The cells were subsequently stained with allophycocyanin-labelled anti-Gr1 mAb and FITC-labelled anti-F4/80 mAb, and then analysed using a FACSCanto II (BD).

### Intracellular Ca^2+^ concentrations

THP-1 cells were treated with LFM-A13 for 30 min and then stimulated with ATP (5 mM) or Leu-Leu-OMe (2 mM) after incubation with the calcium indicator Fluo-4 AM (Invitrogen), according to the manufacturer's instructions. The fluorescence intensity was analysed using a FACSCanto II and FlowJo software (Tree Star).

### Statistical analysis

Statistical analysis of all endpoints was performed using the two-sided Student's *t*-test. The variance among the groups was estimated using the F-test. All data are presented as mean±s.d. *P*<0.05 was considered statistically significant.

## Additional information

**How to cite this article:** Ito, M. *et al.* Bruton's tyrosine kinase is essential for NLRP3 inflammasome activation and contributes to ischaemic brain injury. *Nat. Commun.* 6:7360 doi: 10.1038/ncomms8360 (2015).

## Supplementary Material

Supplementary Information Supplementary Figures 1-12, Supplementary Tables 1-2
