# Supplementary material for: Bruton's tyrosine kinase is essential for NLRP3 inflammasome activation and contributes to ischaemic brain injury
Source: Nat Commun. 2015 Jun 10;6:7360. doi: 10.1038/ncomms8360 (PMC4490404; doi:10.1038/ncomms8360)
Supplement: Supplementary Information — Supplementary Figures 1-12, Supplementary Tables 1-2 [file ncomms8360-s1.pdf]

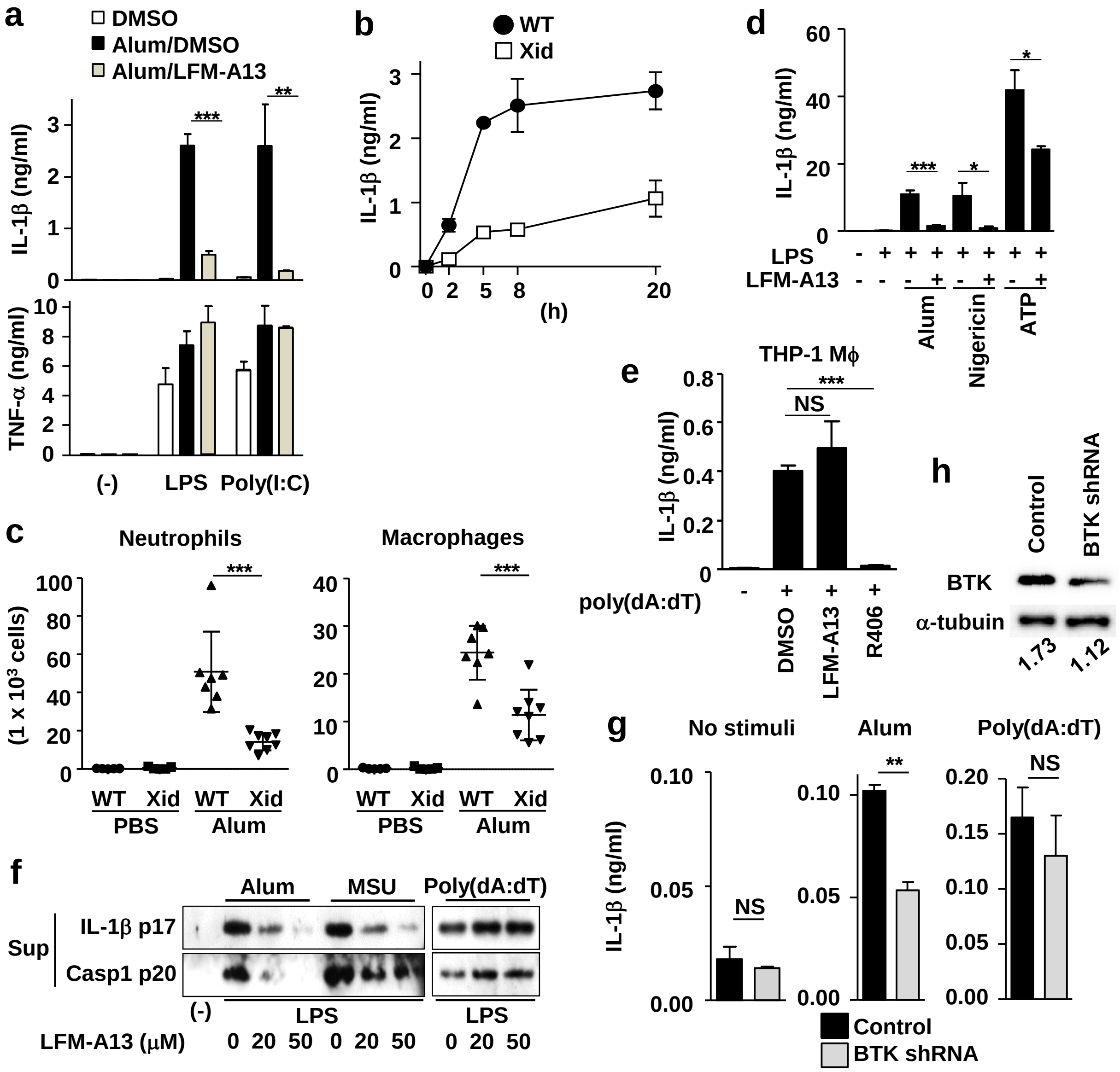

Supplementary Figure 1. BTK is involved in activation of NLRP3 inflammasome, but not AIM2 inflammasome. (**a**) ELISA of murine IL-1β, TNF-α in supernatants of LPS- or poly(I:C)-primed murine peritoneal macrophages that were pretreated with LFM-A13 and then stimulated with alum for 3 h. (**b**) ELISA of murine IL-1β in supernatants of LPS-primed peritoneal macrophages from Xid and WT mice stimulated with alum up to 20 h. (**c**) Flow cytometric analysis of Gr-1^{+}F4/80^{-} neutrophils and F4/80^{+} monocytes-macrophages in the peritoneal cavity from Xid (n = 8) or WT (n = 7) mice that were injected with alum for 15 h. (**d**) ELISA of murine IL-1β in supernatants of LPS-primed BMDCs that were pretreated with LFM-A13 and then stimulated with the indicated NLRP3 inflammasome activators for 3 h. (**e**) ELISA of human IL-1β in supernatants of THP-1-Mϕs that were pretreated with LFM-A13 or R406 and then stimulated with poly(dA:dT) for 3 h. (**f**) Immunoblot analysis of human IL-1β p17 and caspase-1 p20 in supernatants of LPS-primed human blood monocytes that were pretreated with LFM-A13 and then stimulated with the indicated inflammasome activators for 3 h. (**g**) ELISA of human IL-1β in supernatants of BTK-knockdown and control THP-1-Mϕs stimulated without or with alum or poly(dA:dT) for 3 h. Data are representative of three independent experiments. Data are presented as mean ± SD (triplicate). *, $P < 0.03$; **, $P < 0.01$; ***, $P < 0.003$. Two-sided Student's t-test. (**h**) Immunoblot analysis of human BTK and α-tubulin in BTK-knockdown and control THP-1 cells. Lower numbers indicate the protein expression ration of BTK to α-tubulin.

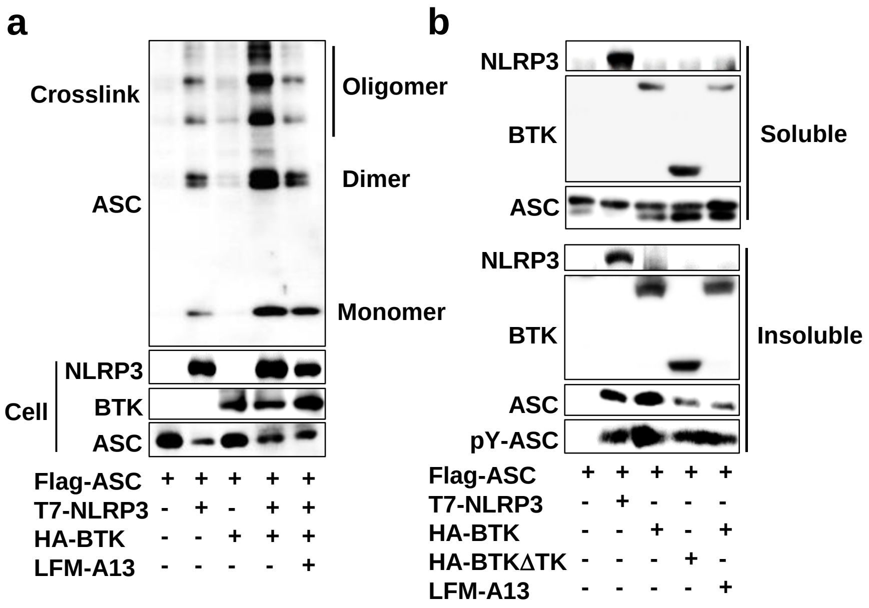

Supplementary Figure 2. BTK overexpression promotes ASC oligomerization and aggregation in HEK 293T cells. (**a**) Immunoblot analysis of human ASC, NLRP3, and BTK in the BS3-treated or -untreated cell lysates of HEK 293T cells that were transfected with Flag-ASC, T7-NLRP3, or HA-BTK. Six hours after the transfection, LFM-A13 was added to the cell culture. (**b**) Immunoblot analysis of human NLRP3, BTK, and ASC in the Triton X-soluble and -insoluble fractions of HEK 293T cells transfected with Flag-ASC, T7-NLRP3, HA-full-length BTK, or HA-BTKΔTK. Six hours after the transfection, LFM-A13 was added to the cell culture. Data are representative of three independent experiments.

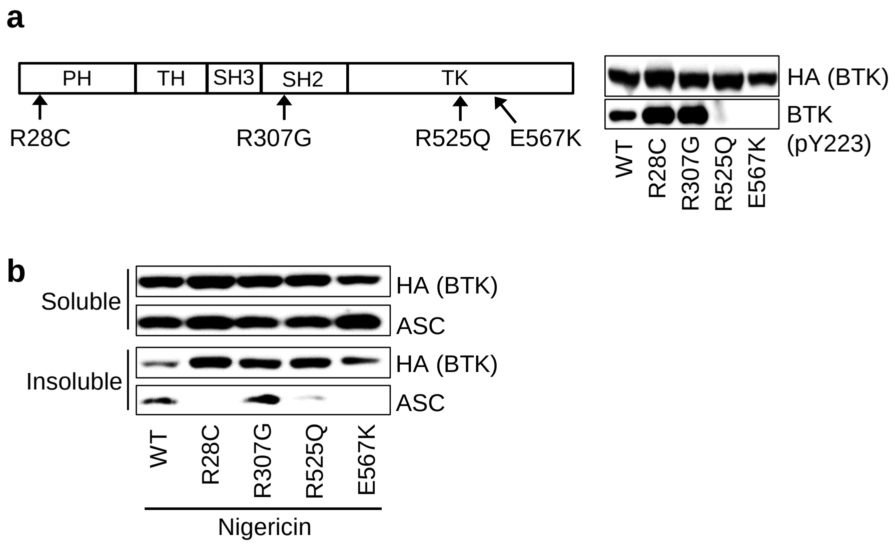

Supplementary Figure 3. XLA-type BTK mutations impede ASC redistribution. (**a**) Schematic of human BTK and four XLA-type mutations (Left) and immunoblot of phosphorylation of Tyr-223 of human BTK in THP-1-Mϕs stably expressing HA-BTK (WT or XLA mutant) (Right). (**b**) Immunoblot analysis of ASC in the Triton X-soluble and -insoluble fractions in THP-1-Mϕs stably expressing HA-BTK (WT or XLA mutant) stimulated with nigericin for 30 min. Data are representative of two independent experiments.

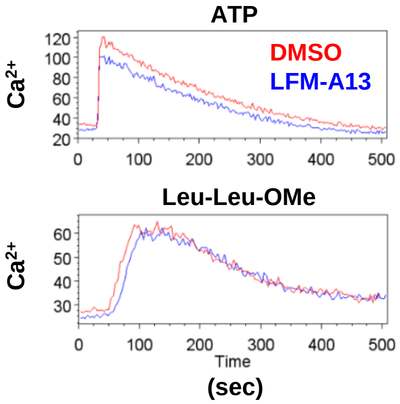

Supplementary Figure 4. LFM-A13 does not inhibit Ca^{2+} influx. Fluo-4 AM-loaded THP-1 cells were treated with LFM-A13, then stimulated with ATP or Leu-Leu-OMe. The fluorescent intensity was measured using flow cytometric analysis. Data are representative of two independent experiments.

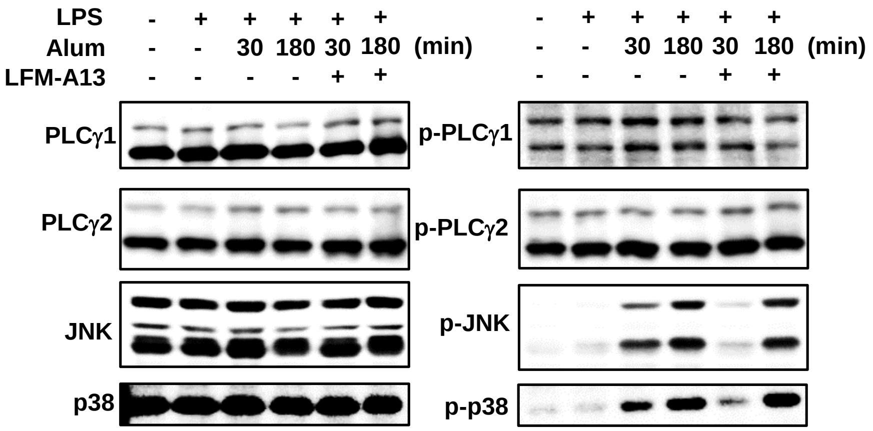

Supplementary Figure 5. LFM-A13 does not inhibit PLCγ phosphorylation. Immunoblot analysis of PLCγ1, PLCγ2, JNK, p38 and the phosphorylated form of each molecules in LPS-primed peritoneal macrophages that were pretreated with LFM-A13 and then stimulated with alum for 30 or 180 min.

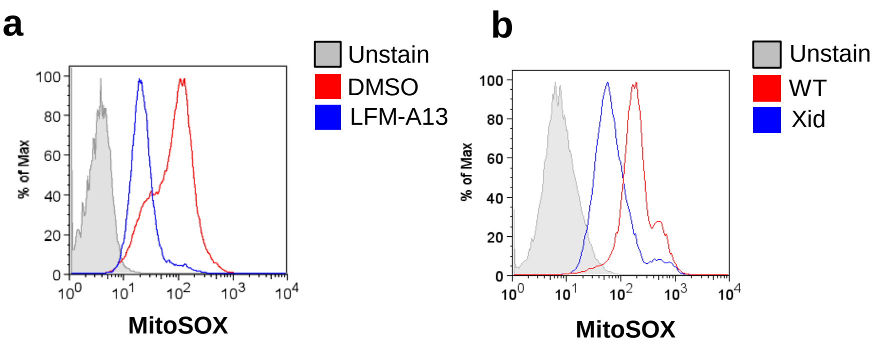

Supplementary Figure 6. BTK is involved in mitochondrial ROS production. MitoSOX-loaded THP-1 cells treated with LFM-A13 (**a**) or peritoneal macrophages from WT (CBA/J) or Xid (CBA/N) mice (**b**) were stimulated with ATP. The fluorescent intensity was measured using flow cytometric analysis. Data are representative of two independent experiments.

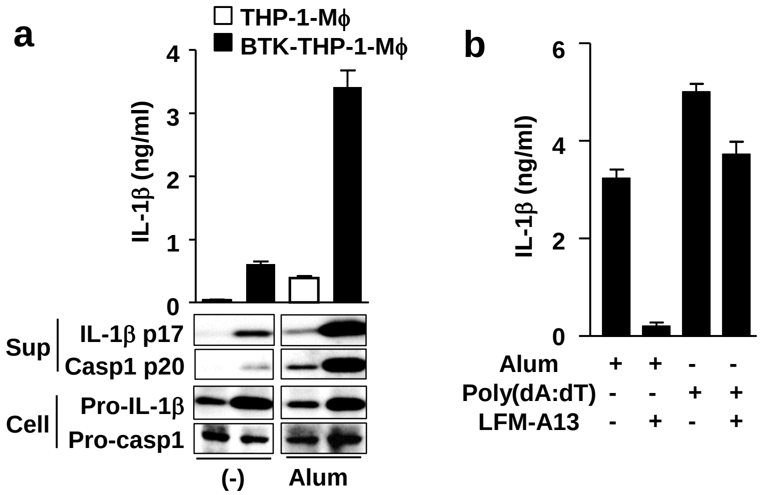

Supplementary Figure 7. BTK overexpression induces NLRP3 inflammasome activation in THP-1-macrophages. ELISA of human IL-1β in supernatants (**a**,**b**) and immunoblot analysis of IL-1β and caspase-1 in supernatants and cell lysates (**a**) of THP-1-Mϕs and THP-1-Mϕs stably expressing HA-BTK that were stimulated with alum or poly(dA:dT) for 3 h. Data are representative of three independent experiments. Data are presented as mean ± SD (triplicate).

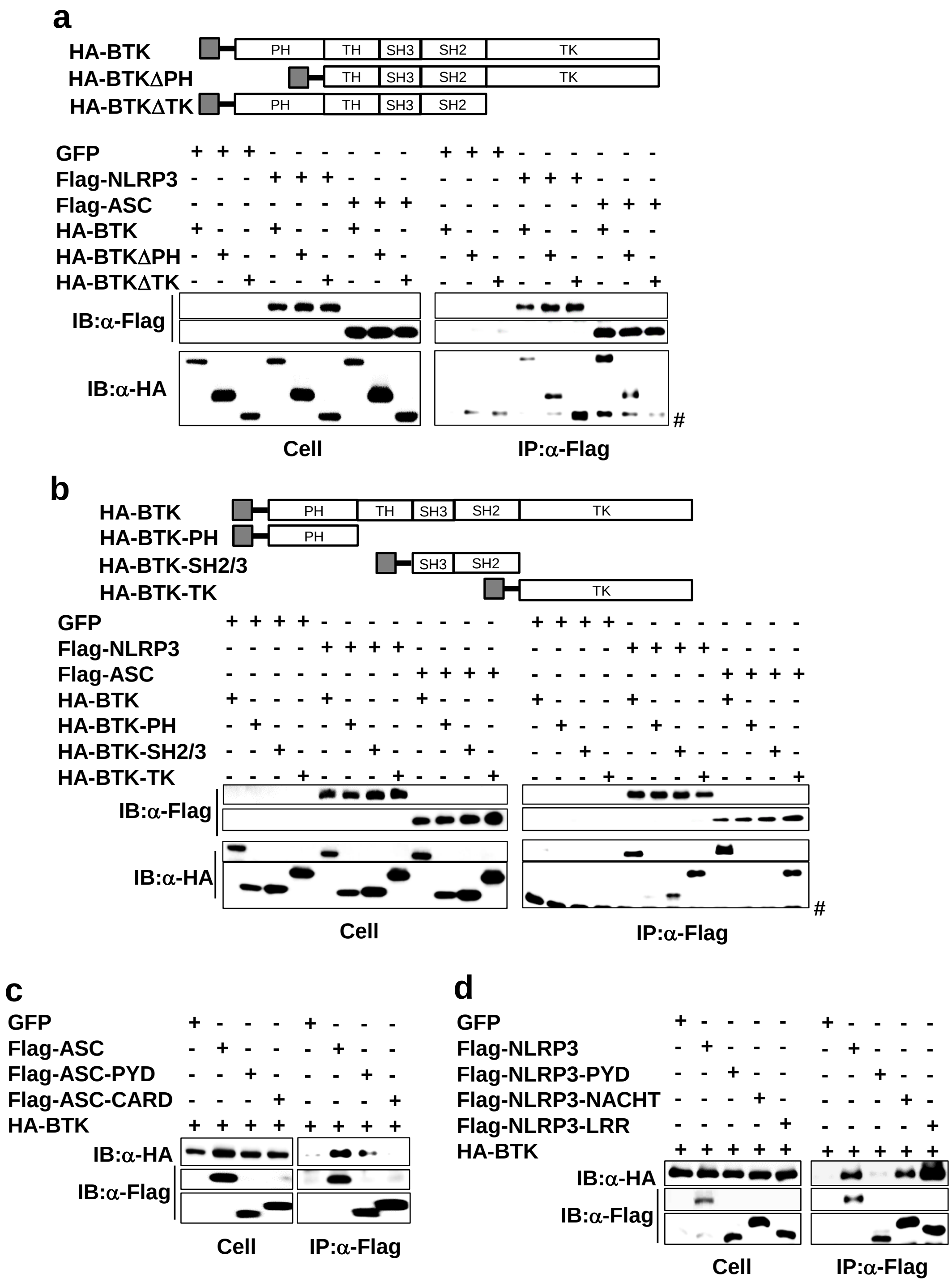

Supplementary Figure 8. Determination of human BTK domains that bind to NLRP3 and ASC. Co-immunoprecipitation and immunoblot assays of HA-BTK (Full-length or ΔPH or ΔTK domains) and Flag-NLRP3 or -ASC (**a**), HA-BTK (Full-length or PH, SH2/3, or TK domains) and Flag-NLRP3 or -ASC (**b**), HA-BTK and Flag- ASC (Full-length, PYD, or CARD) (**c**), or HA-BTK and Flag-NLRP3 (Full-length, PYD, or NACHT or LRR domains) (**d**) from HEK293T cells co-transfected with plasmids expressing the indicated molecules. #, Ig light chains (**a**,**b**). Data are representative of two independent experiments.

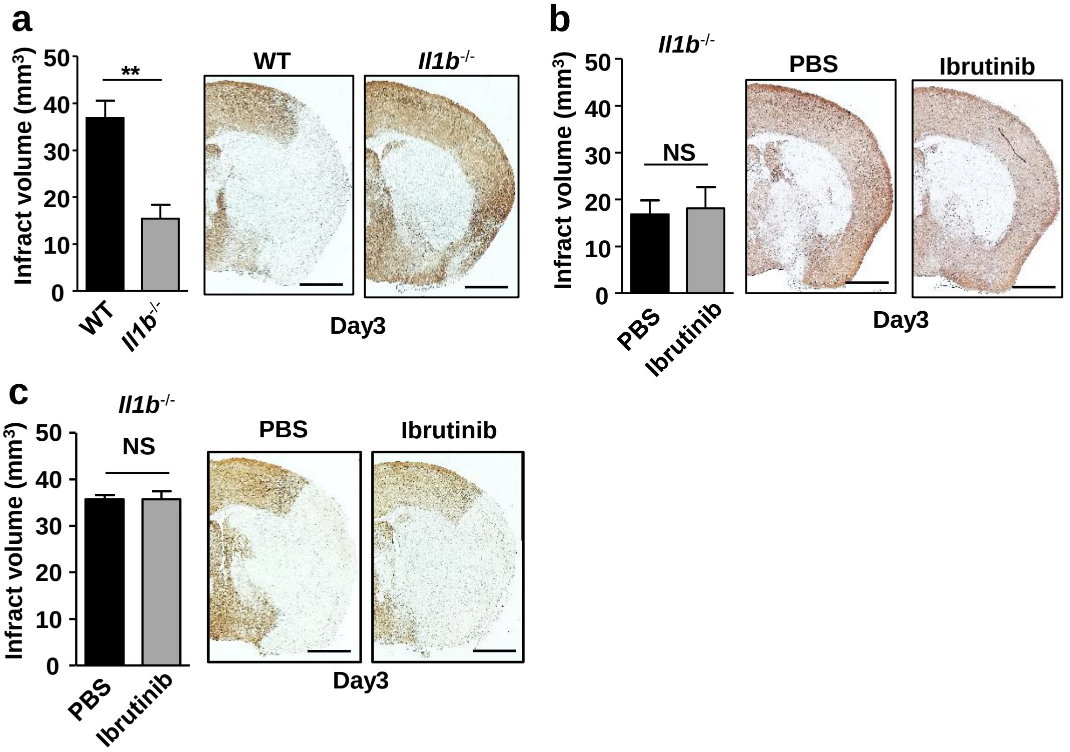

Supplementary Figure 9. IL-1β is responsible for ischemic brain injury. (**a**) Infarct volume on day 3 after stroke onset in WT or *Il1b*$^{-/-}$ mice (n = 8 for WT; n = 7 for *Il1b*$^{-/-}$). (**b, c**) Infarct volume on day 3 after stroke onset in *Il1b*$^{-/-}$ mice treated with PBS or ibrutinib (3.125 mg/kg/day on Day 0, 1) immediately after 60 min (**b**) or 90 min (**c**) occlusion followed by reperfusion (n = 6 for PBS; n = 5 for ibrutinib). Scale bars, 1 mm. Data are presented as mean ± SEM. **, P < 0.01. Two-sided Student's t-test.

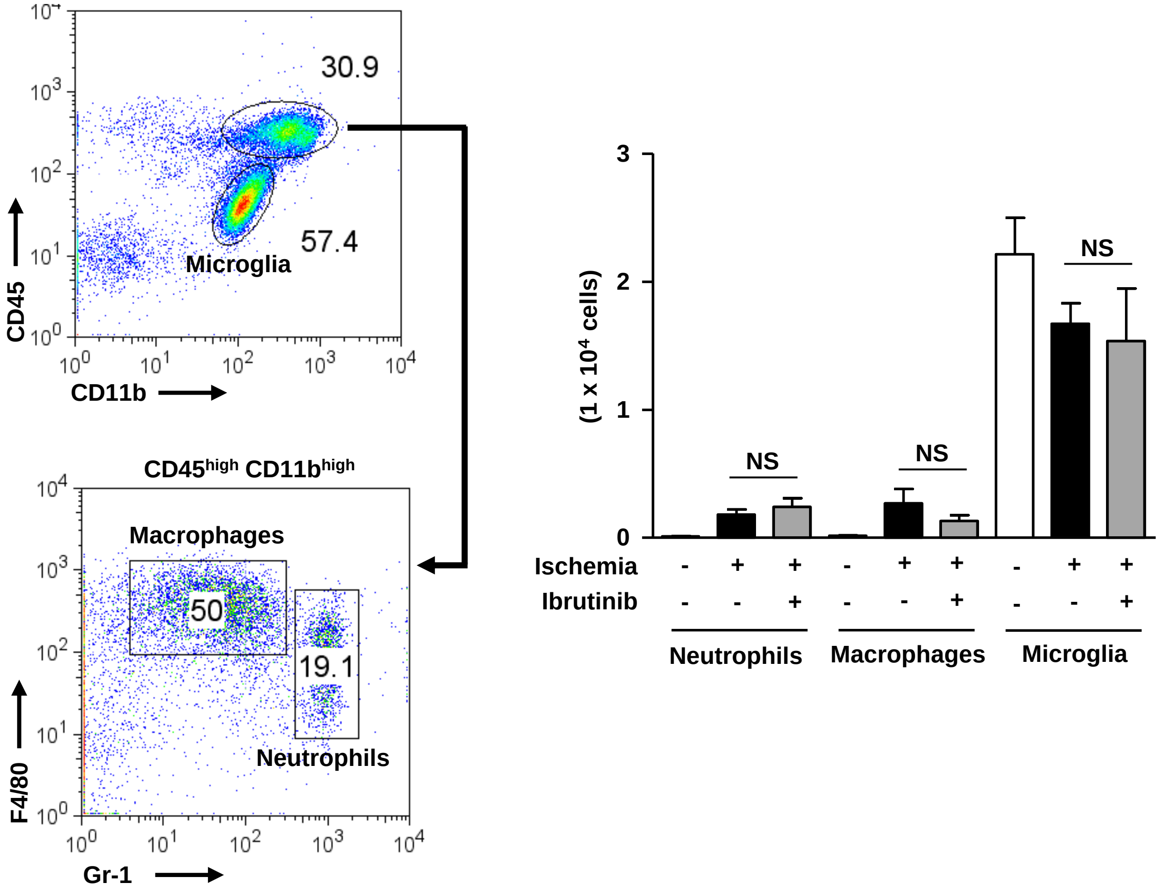

Supplementary Figure 10. The numbers of neutrophils and macrophages are not affected by ibrutinib. The numbers of neutrophils, macrophages, and microglia in the brain were counted by FACS on day 1 after stroke onset (n = 4). Data are presented as mean ± SEM. Two-sided Student's t-test. Left panels show representative FACS profiles of mononuclear cells from the ischemic brain.

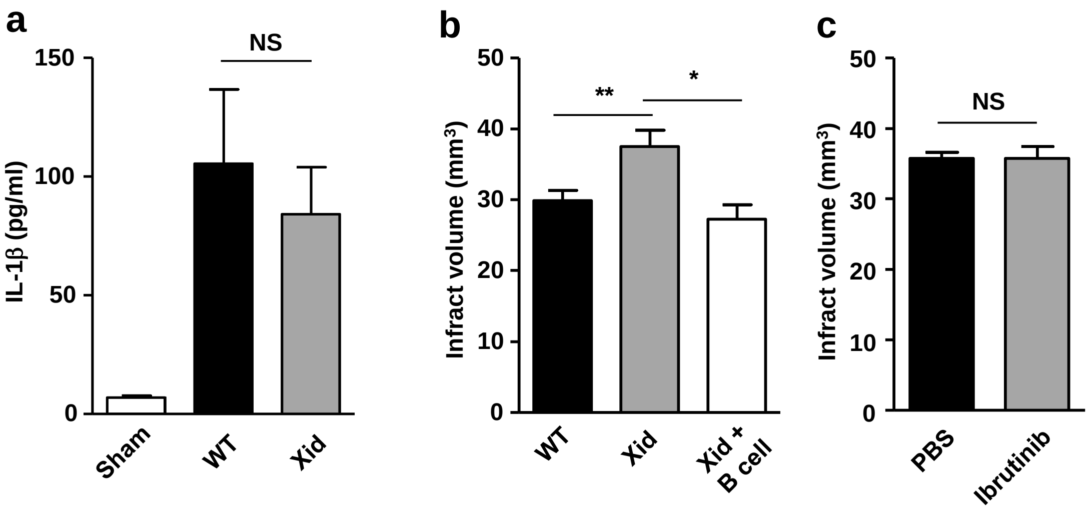

Supplementary Figure 11. Mature IL-1β is reduced in Xid. (**a**) ELISA of IL-1β in brain from WT (CBA/J) or Xid (CBA/N), mice on day 1 after stroke onset (*n* = 3). (**b**) Infarct volume on day 4 after stroke onset in WT, Xid mice, or Xid mice transferred with $CD19^+$ B cells (*n* = 11 for WT; *n* = 8 for Xid; *n* = 4 for Xid + B cell). (**c**) Infarct volume on day 3 after stroke onset in Xid mice treated with PBS or ibrutinib (3.125 mg/kg/day on Day 0) immediately after stroke onset (*n* = 5 for PBS; *n* = 7 for ibrutinib). Data are presented as mean ± SEM. *, $P < 0.03$; **, $P < 0.01$. Two-sided Student's *t*-test.

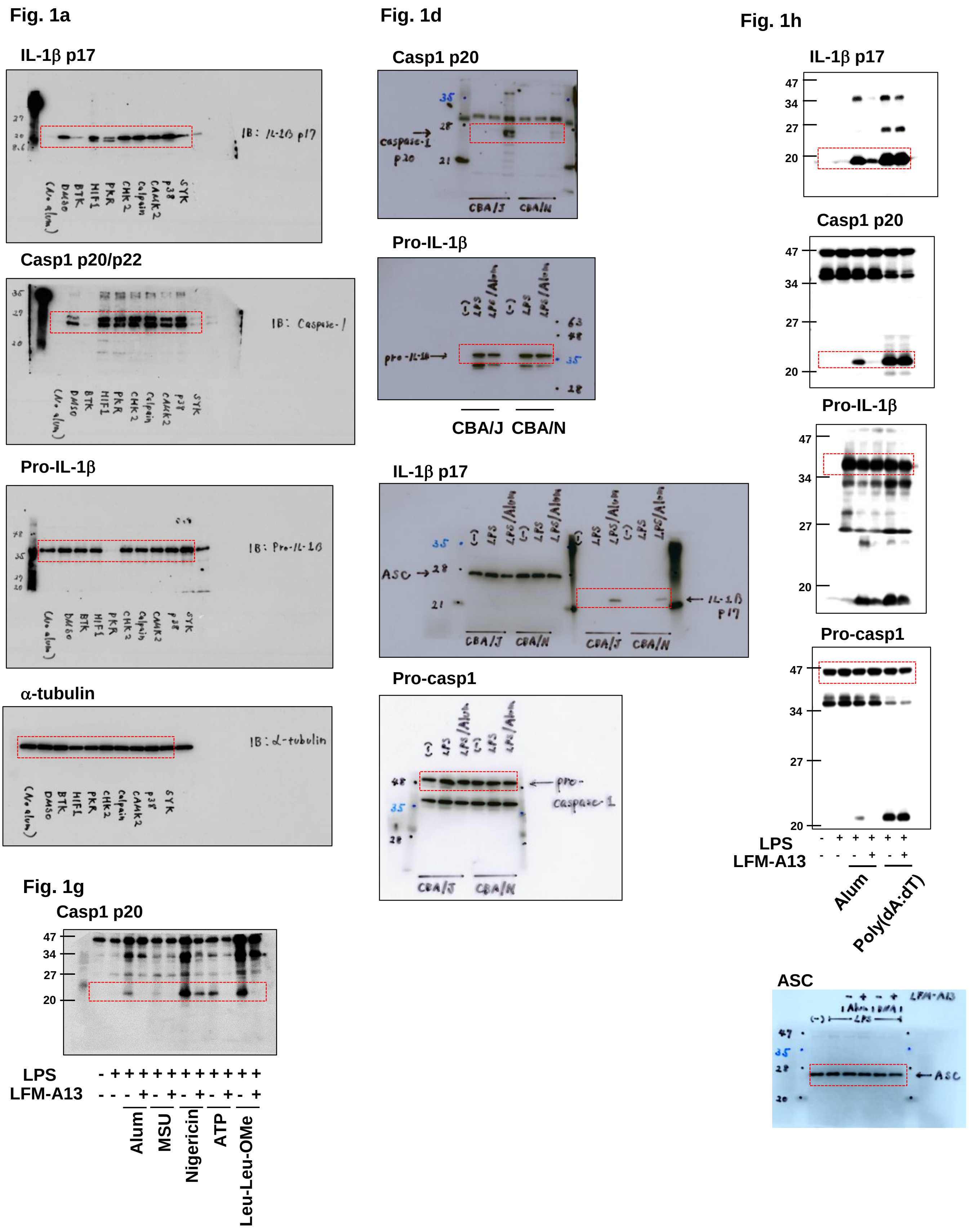

Supplementary Figure 12. Full-length images of Western blots presented in the main figures.

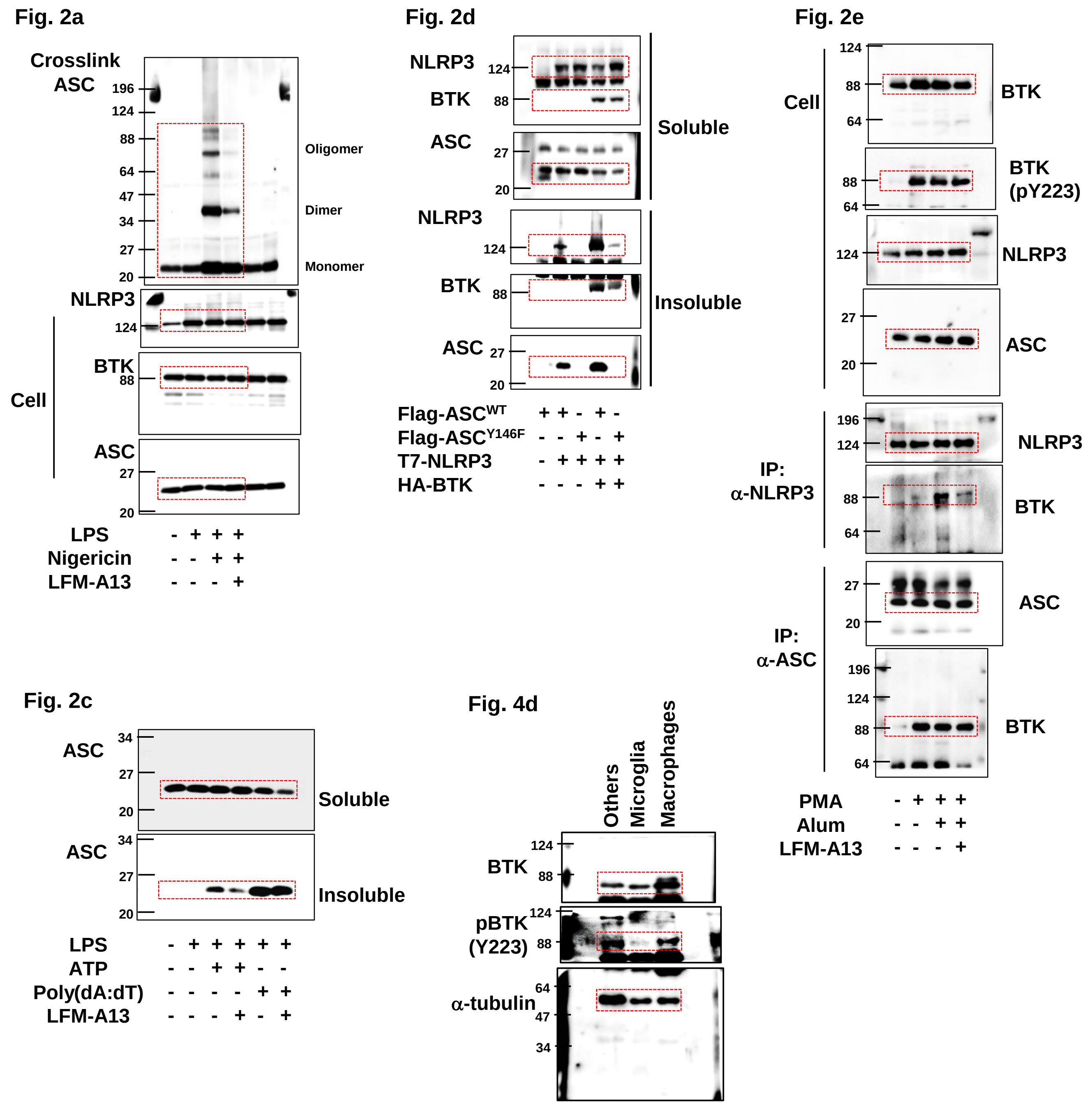

Supplementary Figure 12. Full-length images of Western blots presented in the main figures.

Supplementary Table 1. List of pharmacological inhibitors.

Inhibitor	Company	Target	Final conc. (μM)
LFM-A13	Cayman	BTK	20
Ibrutinib	ShanHai Biochempartner	BTK	10
HIF-1 inhibitor	Calbiochem	HIF1	5
ASN11124542	Calbiochem	PKR	5
Chk2 inhibotr II	Calbiochem	CHK2	2
Calpeptin	Calbiochem	Calpain	5
KN-62	Calbiochem	CAMK2	4
SB203580	Calbiochem	p38	10
R406	ShangHai Biochempartner	Syk	10

Supplementary Table 2. The percentages of cerebral blood flaw reduction after common carotid artery (CCA) and middle cerebral artery (MCA) occlusion (mean ± SD).

		n	Before	After	
				CCA	MCA
WT (C57BL/6J)	PBS	16	100	72±9.6	15.0±2.3
	Ibrutinib	9	100	77.2±9.8	17.0±3.2
Il1b$^{-/-}$ (C57BL/6J)	PBS	13	100	72.7±8.7	16.6±3.6
	Ibrutinib	5	100	75.1±7.6	17.6±1.9
WT (CBA/J)	PBS	8	100	81.9±4.9	16.6±4.2
Xid (CBA/N)	PBS	7	100	77.8±8.1	12.4±4.5
	Ibrutinib	6	100	83.7±8.9	15.1±4.8
	B cell transfer	9	100	78.3±8.2	12.9±4.2
